# Supplementary material for: Integrative Taxonomy Reveals Two New Trichoderma Species and a First Mexican Record from Coffee Soils in Veracruz
Source: J Fungi (Basel). 2025 Dec 1;11(12):856. doi: 10.3390/jof11120856 (PMC12733664; doi:10.3390/jof11120856)
Supplement: Supplementary file 1 [file jof-11-00856-s001.zip › Supplementary material[1].pdf]

Supplementary material

Table 1. Sequences used for molecular phylogenetic analyses of reference sequences from type, ex-type, or vouchered strains of known species within the Harzianum and Virens clades of *Trichoderma* (as of May 2025) and newly generated sequences (in bold).

**Species**	**Voucher/Strain**	**GenBank accession number**		
		**ITS**	**TEF**	**RPB2**
T. achlamydosporum	YMF 1.06226*	MN977791	MT070156	MT052180
T. afarasin	G.J.S. 06-98; CBS 130501	FJ442630	FJ463327	—
T. afarasin	Dis 314f; CBS 130742	FJ442259	FJ463400	FJ442778
T. afroharzianum	G.J.S. 04-186; CBS 124620*	FJ442265	FJ463301	FJ442691
T. aggregatum	HMAS 248863 = CGMCC 3.18406*	KY687946	KY688062	KY688001
T. aggressivum	DAOM 222156 = BPI 748201*	AF443911	AF348098	—
T. aggressivum f. europaeum	CBS 100526*	FJ442607	AF348096	KP009166
T. albidum	YMF 1.7530*	OQ517962	OQ559118	OQ559127
T. albocorneum	G.J.S. 97-28	DQ018116	AY937440	—
T. alni	CBS120633 = C.P.K. 1982*	EU518651	EU498312	EU498349
T. alpinum	HMAS 248821 = CGMCC 3.18385*	KY687906	KY688012	KY687958
T. amazonicum	IB50 = CBS 126898*	HM142358	HM142376	HM142367
T. anaharzianum	YMF 1.00383*	MH113931	MH183182	MH158995
T. asiaticum	YMF 1.00352*	MH113930	MH183183	MH158994
T. atrobrunneum	G.J.S. 92-110; CBS 548.92*	AF443924	AF443942	—
T. atrogelatinosum	CBS 237.63*	MH858272	—	KJ842201
T. auriculariae	JZBQT1Z7*	ON653396	ON649896	ON649949
T. austroindianum	G.J.S. 08-157 = BAFC 3583*	—	MH352421	—
T. azevedoi	CEN 1422*	MK714902	MK696660	MK696821
T. bambusicola	MFLU 18-2747*	ON230047	ON238001	—
T. bannaense	HMAS 248840 = CGMCC 3.18394*	KY687923	KY688037	KY687979
T. botryosum	COAD2422*	—	MK044119	MK044212
T. breve	HMAS 248844 = CGMCC 3.18398*	KY687927	KY688045	KY687983
T. brunneoviride	CBS121130 = C.P.K. 2014*	EU518659	EU498316	EU498357

T. caeruloviride	COAD 2415*	—	MK044109	MK044202
T. camerunense	G.J.S. 99-230; CBS 138272*	AY027780	AF348107	—
T. catoptron	G.J.S. 02-76 = CBS 114232*	AY737766	AY737726	AY391900
T. ceraceum	G.J.S. 95-159 = CBS 114245 = DAOM 232831*	AF275332	AF534603	AF545508
T. ceratophylli	YMF 1.04621*	MK327581	MK327579	MK327580
T. cerinum	DAOM 230012*	—	AY605802	KJ842184
T. christiani	S442 = CBS 132572*	—	KJ665439	KJ665244
T. cinnamomeum	G.J.S. 97-237 = CBS 114235 = DAOM 232833*	AY737759	AY737732	AY391920
T. compactum	YMF 1.01693*	AY941822	AY941824	—
T. concentricum	HMAS 248833 = CGMCC 3.18389*	KY687915	KY688027	KY687971
T. confertum	HMAS 248896*	—	MF371220	MF371205
T. corneum	G.J.S. 97-75 = CBS 100541*	—	AY937431	KJ842183
T. dacrymycellum	WU 29044	FJ860749	FJ860633	FJ860533
T. densissimum	T32434 = CGMCC 3.24126*	—	OP357971	OP357966
T. dichromospora	CBS 337.69*	MH859318	—	—
T. endophyticum	Dis 217a; CBS 130729*	FJ442243	FJ463319	—
T. endophyticum	Dis 217H; CBS 130730	FJ442242	FJ463314	FJ442721
T. endophyticum	**IE7006**	**PV687649**	**PV694681**	**PV694674**
T. epimyces	CBS 120534 = WU 28237*	EU518663	EU498320	EU498360
T. ganodermatigerum	CCMJ5245*	—	ON567195	ON567189
T. globoides	HMAS 248747*	—	KX026955	KX026963
T. gongcheniae	T33441 = GDMCC 3.1011*	—	OR779518	OR779489
T. graminicola	YNE00490 = GDMCC 3.1015*	—	OR779521	OR779494
T. graminis	YNE00410 = GDMCC3.1013*	—	OR779514	OR779491
T. guizhouense	HGUP0038 = CBS 31803*	JN191311	JN215484	JQ901400
T. harzianum	CBS 226.95*	AJ222720	AF534621	AF545549
T. hausknechtii	Hypo 649 = CBS 133493*	—	KJ665515	KJ665276
T. helicolixii	S640 = CBS 133499*	—	KJ665517	KJ665278
T. hengshanicum	HMAS 248852 = CGMCC 3.18402*	KY687935	KY688054	KY687991

T. hirsutum	HMAS 248834 = CGMCC 3.18390*	KY687916	KY688029	KY687972
T. hortense	G.J.S. 08-116 = BAFC 4291*	—	MH253895	—
T. hortense	CEN1243	OM515062	ON101464	—
T. hymenopellicola	GUCC202010*	—	ON102007	ON088661
T. ingratum	HMAS 248822 = CGMCC 3.18386*	KY687917	KY688018	KY687973
T. inhamatum	CBS 273.78*	FJ442680	AF348099	FJ442725
T. italicum	S131 = CBS 132567*	—	KJ665525	KJ665282
T. jaklitschii	CHAX CP61-2*	—	MW480140	MW480149
T. koreanum	SFC20131005-S066 = KACC 48487*	MH050352	MH025979	MH025988
T. lentiforme	DIS 173D	—	FJ851882	FJ442790
T. lentiforme	G.J.S. 98-6; CBS 100542*	AF469189	AF469195	—
T. lentinulae	HMAS 248256 = CGMCC 3.19847*	MN594469	MN605878	MN605867
T. liberatum	HMAS 248831 = CGMCC 3.18388*	KY687913	KY688025	KY687969
T. linzhiense	HMAS 248846*	KY687929	KY688047	KY687985
T. lixii	G.J.S. 97-96; CBS 110080*	AF443920	AF443938	KJ665290
T. longiphialidicum	LESF 552 = CBS 139785 = CBMAI 1827*	KT278901	KT279020	KT278955
T. miyunense	JZBQF7*	—	ON649916	ON649969
T. neoguizhouense	T33324 = GDMCC3.1012*	—	OR779516	OR779487
T. neotropicale	G.J.S. 11-185; CBS 130633*	HQ022407	HQ022771	—
T. paradensissimum	CGMCC3.24125*	—	OP357968	OP357962
T. parapeberdyi	T30677 = GDMCC 3.1016*	—	OR779510	OR779483
T. parepimyces	CBS 122769 = C.P.K. 2421*	FJ860800	FJ860664	FJ860562
T. peberdyi	CEN 1426*	MK714906	MK696664	MK696825
T. peruvianum	CHAX CP15-2*	—	MW480145	MW480153
T. perviride	HMAS 273786*	—	KX026954	KX026962
T. phayaoense	SDBR-CMU349*	MT995122	MW002073	MW002074
T. pholiotae	JZBQH12*	ON653405	ON649919	ON649972
T. pinicola	SFC20130926-S233 = KACC 48486*	MH050354	MH025981	MH025993
T. pleuroti	KCTC 26314 = CBS 124387*	HM142363	HM142382	HM142372

T. pleuroticola	KCTC 26315 = CBS 124383*	HM142362	HM142381	HM142371
T. pollinicola	LC11682 = LF1542 = CGMCC 3.18781*	MF939592	MF939619	MF939604
T. polypori	HMAS 248855 = CGMCC 3.18404*	KY687938	KY688058	KY687994
T. priscilae	S168 = CBS 131487*	—	KJ665691	KJ665333
T. propepolypori	YMF 1.06224*	MN977789	MT070158	MT052181
T. pseudoasiaticum	YMF 1.06200*	MN977792	MT070155	MT052183
T. pseudodensum	HMAS 248828 = CGMCC 3.18387*	KY687910	KY688023	KY687967
T. pseudogelatinosum	TMIC 60186 = TUFC 60186*	JQ797389	JQ797397	JQ797405
T. pseudopyramidale	COAD 2426*	—	MK044131	MK044224
T. purpureum	HMAS 273787*	—	KX026953	KX026961
T. pyramidale	S73 = CBS 135574*	—	KJ665699	KJ665334
T. rifaii	DIS 355b = CBS 130746*	FJ442663	FJ463324	—
T. rufobrunneum	HMAS 252547 = HMAS 244907*	KF729999	KF729992	KF730007
T. rugulosum	SFC20180301-001 = = KACC 48485*	MH050353	MH025984	MH025986
T. sanisidroense	**IE7003**	**PV687650**	**PV694682**	**PV694675**
T. sanisidroense	**IE7004***	**PV687651**	**PV694683**	**PV694676**
T. sanisidroense	**IE7005**	**PV687652**	**PV694684**	**PV694677**
T. shaanxiensis	T32000 = GDMCC3.1014*	—	OR779513	OR779486
T. simile	YMF 1.06201*	MN977793	MT070154	MT052184
T. simmonsii	G.J.S. 91-138 = CBS 130431*	AF443917	AF443935	FJ442757
T. simplex	HMAS 248842 = CGMCC 3.18396*	KY687925	KY688041	KY687981
T. solum	HMAS 248848 = CGMCC 3.18400*	KY687931	KY688050	KY687987
T. stramineum	G.J.S. 02-84 = CBS 114248 = DAOM 232840*	AY737765	AY737746	AY391945
T. subalni	HMAS 275683 = HMAS 247267*	—	MH612377	MH612371
T. syagri	BAFC 4357 = LJC 10690*	—	MG822711	—
T. tawa	G.J.S. 97-174 = CBS 114233 = DAOM 232841*	AY737756	FJ463313	AY391956
T. tenue	HMAS 273785*	—	KX026952	KX026960
T. tomentosum	DAOM 178713a*	DQ085432	AF534630	AF545557

T. velutinum	TUB F784 = DAOM 230013*	AF149873	KJ665769	KF134794
T. vermifimicola	HMAS 248255 = CGMCC 3.19694*	MN594473	MN605882	MN605871
T. viridulum	HMAS 273865*	—	KX026957	KX026965
T. xixiacum	HMAS 248253 = CGMCC 3.19697*	MN594476	MN605885	MN605874
T. zayuense	HMAS 248835 = CGMCC 3.18391*	KY687918	KY688031	KY687974
T. zelobreve	HMAS 248254 = CGMCC 3.19695*	MN594474	MN605883	MN605872
T. zeloharzianum	YMF 1.00268 = CGMCC 3.19082*	MH113932	MH183181	MH158996
T. crassum	DAOM 164916*	EU280067	EU280048	KJ842185
T. crassum	TRS113	KP009300	KP008865	KP009102
T. neocrassum	G.J.S. 01-227 = CBS 114230*	—	JN133572	AY481587
T. jilotepecense	**IE7000***	**PV687646**	**PV694678**	**PV694671**
T. jilotepecense	**IE7001**	**PV687647**	**PV694679**	**PV694672**
T. jilotepecense	**IE7002**	**PV687648**	**PV694680**	**PV694673**
T. virens	Gli 39, ATCC 13213 = CBS 249.59*	AF099005	AF534631	AF545558
T. virens	GJS 01-287	DQ083023	AY750894	EU341804
T. virens	DIS 162	FJ442669	FJ463367	FJ442696
T. ganodermatis	HMAS 248856; CGMCC 3.18405*	KY687939	KY688060	KY687995
T. strictipile	CBS 347.93*	AF400263	AF401324	—
T. strictipile	CBS 347.93 = DAOM 172827*	—	AF534628	AF545555
T. chlamydosporum	HMAS 248850 = CGMCC 3.18401*	KY687933	KY688052	KY687989

*=Type specimen
