# Supplementary figures and images for: Integrative Taxonomy Reveals Two New Trichoderma Species and a First Mexican Record from Coffee Soils in Veracruz
Source: J Fungi (Basel). 2025 Dec 1;11(12):856. doi: 10.3390/jof11120856 (PMC12733664; doi:10.3390/jof11120856)

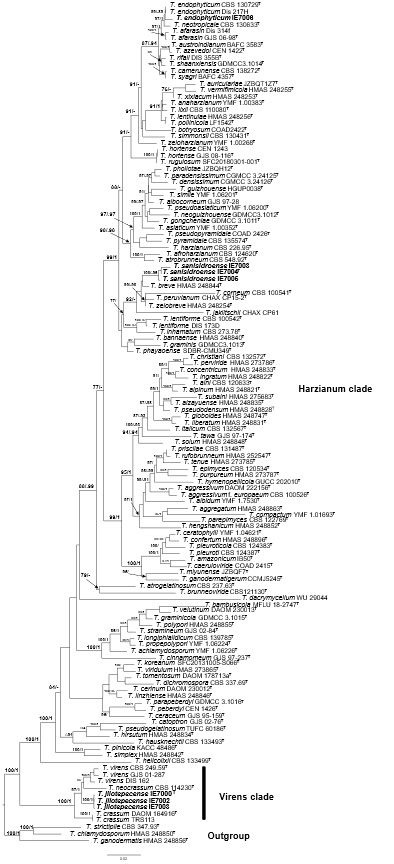

Supplement: Supplementary file 1 [file jof-11-00856-s001.zip › Figure S1.jpg]
